# Exploratory Data Analysis of Cell and Mitochondrial High-Fat, High-Sugar Toxicity on Human HepG2 Cells

**DOI:** 10.3390/nu13051723

**Published:** 2021-05-19

**Authors:** Ricardo Amorim, Inês C. M. Simões, Caroline Veloso, Adriana Carvalho, Rui F. Simões, Francisco B. Pereira, Theresa Thiel, Andrea Normann, Catarina Morais, Amália S. Jurado, Mariusz R. Wieckowski, José Teixeira, Paulo J. Oliveira

**Affiliations:** 1 CNC-Center for Neuroscience and Cell Biology, CIBB-Centre for Innovative Biomedicine and Biotechnology, University of Coimbra, UC-Biotech, Biocant Park, 3060-197 Cantanhede, Portugal; uc2017238707@student.uc.pt (R.A.); velosocaroline@gmail.com (C.V.); uc2012144200@student.uc.pt (A.C.); ruifmsimoes@gmail.com (R.F.S.); jose.teixeira@uc.pt (J.T.); 2 CIQUP/Department of Chemistry and Biochemistry, Faculty of Sciences, University of Porto, 4169-007 Porto, Portugal; 3 PhD Programme in Experimental Biology and Biomedicine (PDBEB), Institute for Interdisciplinary Research (IIIUC), University of Coimbra, 3004-531 Coimbra, Portugal; 4 Laboratory of Mitochondrial Biology and Metabolism, Nencki Institute of Experimental Biology of Polish Academy of Sciences, 02-093 Warsaw, Poland; i.simoes@nencki.edu.pl (I.C.M.S.); m.wieckowski@nencki.edu.pl (M.R.W.); 5 Center for Informatics and Systems, University of Coimbra, Polo II, Pinhal de Marrocos, 3030-290 Coimbra, Portugal; xico@dei.uc.pt; 6 Coimbra Polytechnic-ISEC, 3030-190 Coimbra, Portugal; 7 Mediagnostic, D-72770 Reutlingen, Germany; rhosie1987@gmail.com (T.T.); normann@mediagnost.de (A.N.); 8 Center for Neuroscience and Cell Biology, Department of Life Sciences, University of Coimbra, Calçada Martim de Freitas, 3000-456 Coimbra, Portugal; cattimendesmorais@gmail.com (C.M.); asjurado@bioq.uc.pt (A.S.J.)

**Keywords:** non-alcoholic fatty liver disease (NAFLD), in vitro cell model, Hepg2 cells, lipid accumulation, mitochondria dys(function), exploratory data analysis

## Abstract

Non-alcoholic steatohepatitis (NASH), one of the deleterious stages of non-alcoholic fatty liver disease, remains a significant cause of liver-related morbidity and mortality worldwide. In the current work, we used an exploratory data analysis to investigate time-dependent cellular and mitochondrial effects of different supra-physiological fatty acids (FA) overload strategies, in the presence or absence of fructose (F), on human hepatoma-derived HepG2 cells. We measured intracellular neutral lipid content and reactive oxygen species (ROS) levels, mitochondrial respiration and morphology, and caspases activity and cell death. FA-treatments induced a time-dependent increase in neutral lipid content, which was paralleled by an increase in ROS. Fructose, by itself, did not increase intracellular lipid content nor aggravated the effects of palmitic acid (PA) or free fatty acids mixture (FFA), although it led to an up-expression of hepatic fructokinase. Instead, F decreased mitochondrial phospholipid content, as well as OXPHOS subunits levels. Increased lipid accumulation and ROS in FA-treatments preceded mitochondrial dysfunction, comprising altered mitochondrial membrane potential (ΔΨm) and morphology, and decreased oxygen consumption rates, especially with PA. Consequently, supra-physiological PA alone or combined with F prompted the activation of caspase pathways leading to a time-dependent decrease in cell viability. Exploratory data analysis methods support this conclusion by clearly identifying the effects of FA treatments. In fact, unsupervised learning algorithms created homogeneous and cohesive clusters, with a clear separation between PA and FFA treated samples to identify a minimal subset of critical mitochondrial markers in order to attain a feasible model to predict cell death in NAFLD or for high throughput screening of possible therapeutic agents, with particular focus in measuring mitochondrial function.

## 1. Introduction

Non-alcoholic fatty liver disease (NAFLD) is a worldwide public health concern. The most recent data indicated that 25% of the world population has some form of NAFLD, with the highest prevalence in the Middle East and South America [1]. NAFLD can progress from simple steatosis to non-alcoholic steatohepatitis (NASH), a pathological stage characterized by inflammation and hepatocellular ballooning. Further progression of this condition can result in a fibrotic phenotype (cirrhosis) and, ultimately, in hepatocellular carcinoma (HCC).

NAFLD is well-defined as the augmented accumulation of hepatic triglycerides (TG) (more than 5%) in the absence of excessive alcohol consumption [2] or other liver disease etiology. Over-consumption of high fat-high sugar diets demands a great metabolic effort from the organism, leading to unregulated energy homeostasis processes [2]. Mitochondria are responsible, among other functions, for the oxidation of short and median chain fatty acids through β-oxidation. The deleterious sequence of cellular events includes fatty acid and/or carbohydrates overload followed by an increased reactive oxygen species (ROS) production, mitochondrial damage, and endoplasmic reticulum (ER) stress, resulting in activation of either pro-survival or pro-apoptotic pathways.

Several in vitro models are used to investigate NAFLD mechanisms based on fatty-acid overload. Those different models significantly differ in nature, amount, and fatty acids (FA) overload incubation time, making the data difficult to compare. Although lipotoxicity resulting from incubating in vitro models with high concentrations of lipids and lipid derivatives exposure has been reported in several in vitro studies [3,4], different cellular outcomes were attained depending on lipid composition and/or level of unsaturation, which suggests different cellular responses depending on FA composition and incubation time. For example, palmitic acid (PA; 16:0)-treated H4IIE liver cells displayed increased ER stress, caspase-3 activity, and liver injury [5,6]. The cellular pathways underlying this process involve activation of c-jun NH2-terminal kinase (JNK) and Bcl-2 Homology 3 (BH3)-only protein-induced mitochondrial and lysosomal dysfunction [7,8,9,10,11]. In vitro studies involving monosaturated fatty acids (MUFA) treatment in hepatocytes, such as oleic acid (OA, 18:1), reported a more severe hepatic steatosis when compared with PA exposure [4]. Still, oleic acid treatment showed protective effects against PA-induced toxicity [4,12]. An increase in peroxisome proliferator-activated receptor (PPAR) β/δ expression dependent on the activation of free fatty acid receptor 1 (FFAR1) and a calcium-dependent mechanism was observed in human hepatocytes treated with oleic acid [13]. Moreover, polyunsaturated fatty acids (PUFA) also showed beneficial outcomes in several NAFLD models [14,15]. Long-chain ω-3 PUFAs, especially icosapentaenoic acid (EPA) and docosahexaenoic acid (DHA), presented potent anti-inflammatory activity and anti-fibrosis effect by down-regulated the protein level of YAP/TAZ and, consequently, inhibition of hepatic stellate cells (HCS) activation and proliferation [16]. More recently, studies with bioactive branched fatty acid esters of hydroxyl fatty acids (FAHFAs) showed protective effects against FA-induced mitochondrial dysfunction and intracellular lipid accumulation in HepG2 cells and primary hepatocytes (PMH) [17].

Another newer paradigm on NAFLD metabolic syndrome strongly suggested that carbohydrates-rich diets also play a vast, if not determinant, role in NAFLD development [18,19]. The excess of carbohydrates (e.g., fructose) derived from these diets can be converted into FFA and TG in the liver, also resulting in an increased accumulation of hepatotoxic lipids such as lysophosphatidylcholine (LPC), ceramides, free cholesterol, and bile acids (BA) [20,21,22,23,24]. Fructose-rich diets are recognized to cause steatosis, dyslipidemia, insulin resistance, and liver fibrosis in animal and human studies [25,26].

FA and/or sugars overload within cells leads to a significant increase in lipid accumulation in the cytoplasm, which can be toxic if high concentration levels are reached [27]. The multiplicity of models and lipid (and sugar)- induced toxicity makes an accurate analysis of the role of mitochondrial dysfunction in the progression of cellular damage in vitro difficult to characterize. In this context, the objectives of this work were to investigate in human HepG2 cells (a) how mitochondrial disruption develops under different lipotoxicity protocols, (b) whether mitochondrial dysfunction precedes oxidative stress and induction of cell death or is instead a consequence of those two events and (c) whether fructose (F) worsens the cellular lipotoxicity profile under the different protocols. For these objectives, we characterized and compared the time-dependent cellular effects of different FA overload strategies in the presence or absence of carbohydrates (fructose), with a particular focus on measuring mitochondrial structure, integrity, and bioenergetics, to attain a feasible and reproducible in vitro model for study mitochondrial impairment during progressive cellular lipotoxicity. A suite of computational data analysis tools was applied to several cellular and mitochondrial markers, aiming at identifying differences in the effects of the PA and FFA treatments. Unsupervised learning methods were applied to identify clear separations between groups of samples, corresponding to different cell treatments. The partition was performed with a minimal subset of experimental endpoints. This computational approach is thus an innovative tool for clustering biological effects of supra-physiological lipid levels.

## 2. Material and Methods

### 2.1. Chemicals

Dulbecco’s modified Eagle’s medium (DMEM), penicillin, streptomycin, fetal bovine serum (FBS) and Trypsin were purchased from Gibco-Invitrogen (Grand Island, NY, USA). Dithiothreitol (DTT), phenylmethanesulfonyl fluoride (PMSF), protease inhibitor cocktail (leupeptin, antipain, chymostatin, and pepstatin A), sulforhodamine B (SRB), palmitic acid (C16:0), stearic acid (C18:0), oleic acid (C18:1), linoleic acid (C18:2), arachidonic acid (C20:4), D(-)-Fructose, Nile Red, Hoechst 33342, tetramethylrhodamine methyl ester (TMRM), and 5-(and-6)-chloromethyl-2′,7′-dichlorodihydrofluorescein diacetate, acetyl ester (CM-H_2_DCFDA) were purchased from Invitrogen (Eugene, OR, USA).

### 2.2. Fatty Acid Preparation

Palmitic acid (PA)/Bovine Serum Albumin (BSA, Catalogue N°: A6003, Sigma-Aldrich, St. Louis, MO, USA) solution was prepared by mixing free-fatty acid BSA (0.2 g/mL) with 10 mM palmitic acid in the proportion of 1:1 during 1 h at 37 °C. The Free-fatty acid BSA (0.2 g/mL) was diluted in the same proportion with 150 mM NaCl and used as a control. The fatty acid mixtures (FFA) were prepared as saponified 10 mM stock solutions and complexed (1:1) with free-fatty acid BSA (10 min at 50 °C), cooled to room temperature. The free-fatty acid BSA (0.2 g/mL) was diluted in the same proportion with 25 mM KOH and used as a control. The same ratios of FA dilutions were used for free fatty acid BSA control. The amounts of the different FA in the mixture were: 39% C16:0 (Catalogue N°: P0500, Sigma-Aldrich, St. Louis, MO, USA); 5% C18:0 (Catalogue N°: 85679, Sigma-Aldrich, USA); 50% C18:1 (Catalogue N°: O1008, Sigma-Aldrich, St. Louis, MO, USA); 4% C18:2 (Catalogue N°: L1376, Sigma-Aldrich, USA); 2% C20:4 (Catalogue N°: A3611, Sigma-Aldrich, St. Louis, MO, USA) [28,29].

### 2.3. Cell culture and FA Treatments

Human hepatocellular carcinoma HepG2 cells (Catalogue N°: 85011430, ECACC, Porton Down, UK) were cultured in low-glucose medium composed by Dulbecco’s modified Eagle’s medium (DMEM; Catalogue N°: D5030, Sigma-Aldrich, St. Louis, MO, USA) supplemented with 5 mM glucose, sodium bicarbonate (3.7 g/L), HEPES (1.19 g/L), L-glutamine (0.876 g/L), sodium pyruvate (0.11 g/L), 10% fetal bovine serum (FBS), 1% penicillin-streptomycin 100x solution in a humidified atmosphere (5% CO_2_, 37 °C). HepG2 cells were seeded (6 × 10^4^ cells/cm^2^) and grown for 24 h during which they reached 60–70% confluence before treatment. Next, cells were incubated in the presence of vehicle (BSA 0.01 g/mL) and different mixtures of FA (PA, 0.5 mM or FFA, 0.25 mM) in the presence or absence of fructose (F, 10 mM) for 1, 6 and 24 h.

### 2.4. Nile Red Staining

Cells were seeded in 96-well plates and then subjected to the different treatments. After incubation, the neutral lipid accumulation was assessed through the Nile Red assay [30]. Briefly, the cell culture medium was discarded and 100 µL of the Nile red solution was added to each well for 1 h/1.5 h in the dark conditions at 37 °C. Nile Red is freshly diluted 1:200 in medium without FBS from the stock solution (stock: 0.5 mg/mL dissolved in acetone). Nile Red was then removed, and cells were washed twice with PBS 1X. The fat content per well (in 100 µL PBS 1X) was measured fluorimetrically with 520 nm excitation and 620 nm emission in a Biotek Cytation 3 reader (Biotek Instruments, Winooski, VT, USA). Results were normalized for cell mass content at the end of the assay, using the SRB method [31].

### 2.5. Phospholipid Analysis

HepG2 cells were seeded in 100 mm cell culture dish and submitted to the different treatments. After incubation time, cells were harvested and washed with cold PBS 1X. In order to obtain total cellular extracts, cells were harvested, washed with cold PBS 1X, and then two centrifugation steps were performed for 5 min at 1000× *g* (4 °C). To obtain mitoplast fractions, digitonin (4 mg/mL) was added and kept on ice for 10 min. The resuspension was further diluted with PBS 1X and centrifuged for 10 min, 4 °C, 10,000× *g*. The process was repeated two more times with a 5 min centrifugation at 4 °C, 10,000× *g*. Mitoplasts were stored at −80 °C until further processing. Mitochondrial lipids were extracted accordingly to Bligh and Dyer method [32]. Lipids quantification was performed according to the phosphorous assay described by Rouser et al. [33]. The method was calibrated with a standard curve with known concentrations of KH_2_PO_4_. Pre-washed Whatmann LK5 thin-layer plates with chloroform/methanol (1:1) were sprayed with 1.8% of boric acid solution, air-dried, and then activated for 15 min at 100 °C. A total of 40 µg of phospholipid samples were loaded in the concentration zone of the plates. After, plates were placed in chromatography tanks with a mixture of chloroform/ethanol/water/trimethylamine (30/35/7/35, *v*/*v*) for 2 h. Phospholipids bands were then visualized by soaking the plate in 10% (*m*/*v*) cupric sulfate/8% (*v*/*v*) phosphoric acid solution and just after, plates were heated in an oven at 140 °C for 20 min. Different phospholipids classes were photographed with Biospectrum—Multispectral imaging system (UVP; LLC Upland, CA; Cambridge, UK). The densities of each band were calculated with TotalLab TL120 1D v2009.

### 2.6. Intracellular Oxidative Stress

Cells were seeded in a 96-well plate and then subjected to the different treatments. After incubation time, intracellular ROS levels were assessed using the oxidative stress-sensitive report molecule CM-H_2_DCFDA (5-(and-6)-chloromethyl-2′,7′-dichlorodihydrofluorescein diacetate, acetyl ester) (Life Technologies, Invitrogen, Carlsbad, CA, USA). Briefly, cells were loaded with 5 μM of CM-H_2_DCFDA in assay buffer (NaCl 120 mM, KCl 3.5 mM, NaHCO_3_ 5 mM, NaSO_4_ 1.2 mM, KH_2_PO_4_ 0.4 mM, HEPES 20 mM supplemented with CaCl_2_ 1.3 mM, MgCl_2_ 1.2 mM and sodium pyruvate 10 mM, pH 7.4) at 37 °C and 5% CO_2_ in the dark for 15 min. Then, cells were washed twice with PBS 1X, and fluorescence signals were measured with 520 nm excitation and 620 nm emission wavelengths using a microplate reader (Cytation 3; BioTek US, Winooski, VT, USA). Results were normalized for cell mass content at the end of the assay, using the SRB method.

### 2.7. Mitochondrial Morphology Imaging

Vital confocal fluorescent microscopy was performed to visualize alterations in mitochondrial electric potential polarization and network distribution in HepG2 cells. Cells were seeded in pre-coated (collagen I 0.15 mg/mL) µ-Slide 8 well ibiTreat (2 × 10^4^/cm^2^) (Ibidi, Gräfelfing, Germany) with a final volume of 300 µL per well, and then subjected to the different treatments. Cells were incubated with fluorescent dyes tetramethylrhodamine (TMRM) (100 nM) and Hoechst 33,342 (1 µg/mL) for mitochondrial network and nuclei, respectively, 30 min before the end of the treatment time in fresh cell culture medium at 37 °C and 5% CO_2_ in the dark conditions. Images were acquired using a Laser Scanning Confocal Microscope (LSM 710, Zeiss, Dublin, CA, USA) equipped with a α-Plan-Apochromat 63x/1.4 Oil DIC M27 objective (Zeiss) and analyzed with ImageJ Fiji program (Scion Corporation, Chicago, IL, USA). Index of interconnectivity was quantified by using a Mitochondria Morphology Macro [34].

### 2.8. Mitochondrial DNA Copy Number Measurements

Mitochondrial DNA copy number was measured using quantitative polymerase chain reaction (qPCR). HepG2 cells were seeded in 60 mm cell culture dish and subjected to the different treatments. Cells were then harvested at the time-points indicated by aspirating media and washing plates with ice-cold PBS. For measurement of mitochondrial DNA copy number, RNase-treated total DNA was isolated using the Qiagen DNeasy kit (Catalogue N°: 69104, Qiagen, Germany) according to the manufacturer’s recommendations. DNA amount and purity were evaluated in a NanoDrop 2000 spectrophotometer (ThermoScientific, Waltham, MA, USA). qPCR was performed based on the amplification of *cytochrome B* (*CytB*) (encoded on the mitochondrial genome; variable quantity in each cell) and *beta-2-microglobulin* (*b2m*) (encoded on the nuclear genome; fixed quantity in each cell) using a Roche Light Cycler and Roche FastStart DNA Master SYBR Green protocols. Human primers for *Cyt B* were: forward 5′-CCACCCCATCCAACATCTCC-3′, reverse 5′-GCGTCTGGTGAGTAGTGCAT-3′ (Pair rating: 66,1; Product length: 112); primers for *b2m* were: forward 5′-GAATTCCAAATTCTGCTTGCTTGC-3′, reverse 5′-CCTCTAAGTTGCCAGCCCTC-3′ (Pair rating: 71.2; Product length: 199). Each reaction was performed in triplicate with an efficiency between 90 and 110%. For amplification purposes, total DNA (25 ng) went on an initial cycle of 2 min at 95 °C, followed by 40 cycles of 5 s at 95 °C plus 20 s at 63 °C and 20 s at 72 °C. At the end of each cycle, Eva Green fluorescence was recorded to enable the determination of Cq. Several dilutions of the control sample and DNA-free water were used as standards and negative control, respectively. The specificity of each reaction for a single product was verified by melting analysis. The cycle number of linear amplifications for each sample was compared with the five-point standard curve to determine the number of template copies present at the start of each reaction. Mitochondrial copy number was estimated by the number of copies of *cytochrome B* template divided by the number of copies of *beta-2 microglobulin* template. The reactions were performed on a CFX™96 Real-Time system (Bio-Rad, Hercules, CA, USA). The normalized expression was calculated by the comparative quantification algorithm ∆∆Ct (CFX Manager™ 3.1 software, 18 Bio-Rad).

### 2.9. Western Blotting Analysis

Cells were seeded in 100 mm cell culture dish and then subjected to the different treatments. In order to obtain total cellular extracts, cells were harvested, washed with cold PBS 1X, and then two centrifugation steps were performed for 5 min at 1000× *g* (4 °C). Cellular pellet was resuspended in cell RIPA buffer (50 mM Tris pH 8, 150 mM NaCl, 5 mM EDTA, 15 mM, MgCl_2_ and 1% Triton X-100) supplemented with 0.5 mM phenylmethylsulfonyl fluoride (PMSF), protease inhibitor cocktail (PIC) (Sigma P8340), 20 mM sodium fluoride (NaF), 5 mM sodium butyrate, 10 mM NAM, DOC 10% and keep on ice for 30 min. After that, the resuspension was mixed and centrifuged at 20,000× *g* for 10 min. Soluble protein contents were determined by the BCA method using BSA as a standard. Laemmli buffer (from Bio-Rad) was added to the samples. An equal amount of proteins (20–50 μg) was separated by electrophoresis on 12% SDS–polyacrylamide gels (SDS–PAGE) and electrophoretically transferred to a polyvinylidene difluoride (PVDF) membrane. After blocking with 5% milk in TBST (50 mM Tris–HCl, pH 8; 154 mM NaCl and 0.1% tween 20) for 2 h at room temperature, membranes were incubated overnight at 4 °C with the antibodies directed against the denatured form of OXPHOS complexes cocktail (1:1000; ab110411, Abcam, Cambridge, UK), Ketohexokinase (1:500, sc-377411, Santa Cruz Biotechnology,Dallas, TX, USA) and β-Actin (1:5000; MAB1501, Chemicon international, Temecula, CA, USA). Membranes were further incubated with goat anti-mouse IgG (1:5000, CS7076, Cell Signaling Technology, Danvers, MA, USA) and goat anti-rabbit IgG (1:5000, CS7074, Cell Signaling Technology, USA) secondary antibodies for 1 h at room temperature. Membranes were then incubated with the ECL detection system (Bio-Rad 1705061) and imaged with the Biospectrum—Multispectral imaging system (UVP; LLC Upland, CA; Cambridge, UK). The densities of each band were calculated with TotalLab TL120 Software (version 2009).

### 2.10. BN-PAGE in-Gel Activity of Complex I

HepG2 cells were seeded in 100 mm cell (6 × 10^4^/cm^2^) culture dish and submitted to the different treatments. After 6 and 24 h, cells were harvested and washed with cold PBS 1X. Cell pellets were resuspended in cold PBS 1X. To obtain mitoplast fractions, digitonin (4 mg/mL) was added and kept on ice for 10 min. The resuspension was further diluted with PBS 1X and centrifuge during 10 min, 4 °C, 10,000× *g*. Two washings with PBS1x for 5 min (4 °C, 10.000× *g*) were performed. When not used on the day, pellets were storage at −80 °C. OXPHOS complexes isolation was attained by adding ACBT buffer (1.5 M epsilon-aminocaproic acid, 75 mM Bis-Tris, pH 7.0), 20% lauryl maltoside, and kept on ice during 10 min. Afterward, samples were centrifuged for 30 min (4 °C, 10,000× *g*) and the supernatant collected. Protein content was assayed by BCA method using BSA as standard. Pre-cast gels with a gradient concentration of 3–12% were loaded with 10 μg of protein-containing BN-sample buffer 1:10 (750 mM aminocaproic acid, 50 mM Bis-Tris, 0.5 mM EDTA, 5% Serva Blue G, pH 7.0) [35]. Gel ran with a constant voltage at 75 V for 30 min. Next, cathode blue buffer was replaced by BCA method cathode light buffer. The voltage was increased up to 150 V until the samples reached the bottom of the gel. After electrophoresis, gels were further processed for in-gel activity assays. Complex I: 3 mM Tris–HCl, pH 7.4, 80 µg/mL NADH, and 0.2 mg/mL nitro tetrazolium blue (NTB)**.** For Complex I activity, gels were incubated for 6 and 24h at 37 °C. Complex activity was scanned or photographed with Biospectrum-Multispectral imaging system (UVP; LLC Upland, CA; Cambridge, UK). The densities of each band were calculated with Image Studio Lite 5.2 Software (LI-COR Biosciences, Lincoln, NE, USA).

### 2.11. Cellular Oxygen Consumption Rate Measurements

Cells were seeded in 96-well plate (pre-coated with collagen I 0.15 mg/mL) under the same conditions described above at a density of 10,000 cells/100 µL/well. After incubation time, oxygen consumption was measured at 37 °C using a Seahorse XFe96 Extracellular Flux Analyzer (Agilent Scientific Instruments, Santa Clara, CA, USA). In addition, an XFe96 sensor cartridge for each cell plate was placed in a 96-well calibration plate containing 200 µL/well calibration buffer and left to hydrate overnight at 37 °C. The cell culture medium from the plates was replaced the following day with 175 µL/well of pre-warmed low-buffered serum-free minimal DMEM medium (D5030, Sigma-Aldrich, St. Louis, MO, USA), the pH adjusted to 7.4 and incubated at 37 °C for 1 h to allow the temperature and pH of the medium to reach equilibrium before the first-rate measurement. Oligomycin, carbonyl cyanide-4-(trifluoromethoxy)phenylhydrazone (FCCP), rotenone, and antimycin A were prepared in DMSO.

For oxygen consumption rate (OCR) measurements, 2 µM oligomycin, injected into reagent delivery port A, 0.33 µM FCCP injected into port B, 1 µM rotenone and 1µM antimycin A injected into reagent delivery port C were diluted in low-buffered serum-free DMEM medium and the pH adjusted to 7.4 with 1 M NaOH. 25 µL of compounds was then pre-loaded into the ports of each well in the XFe96 sensor cartridge. The sensor cartridge and the calibration plate were loaded into the XFe96 Extracellular Flux Analyzer for calibration. When the calibration was complete, the calibration plate was replaced with the study plate. Three baseline rate measurements of OCR and extracellular acidification rate (ECAR) of the HepG2 cells were made using a 3 min mix, 5 min measuring cycles. The compounds were then pneumatically injected by the XFe96 Analyzer into each well, mixed, and OCR measurements made using a 3 min mix, 5 min measuring cycles. Results were analyzed by using the Software Version Wave Desktop 2.6.

### 2.12. Measurement of Caspase 8 and 9-Like Activities

Cells were seeded in 100 mm cell culture dish and then exposed to the different treatments. To obtain total cellular extracts, cells were harvested, washed with cold PBS 1X, and then two centrifugation steps were performed for 5 min at 1000× *g* (4 °C). Floating cells were also collected and combined with adherent cells. Cellular pellets were resuspended in cell lysis buffer supplemented with 100 µM PMSF, 2 mM DTT. The resuspension was homogenized by 30 passages through a 27-gauge needle, followed by 3 cycles of freeze/thaw in liquid nitrogen, and kept at −80 °C until used. Protein content was determined by the Bradford method [31], using BSA as standard. To measure caspase 9-like activity, aliquots of cell extracts containing 100 μg of total protein were incubated in a reaction buffer containing 10% sucrose, 10 mM dithiothreitol (DTT), 0.1% 3[(3-cholamidopropyl) dimethylammonio]-propanesulfonic acid, 25 mM HEPES (pH 7.4) and 100 μM caspase substrate (Ac-LEHD-pNA) for 2 h at 37 °C. Caspase-like activity was determined by following the appearance of the chromophore pNA after cleavage from the labeled substrate Ac-LEHD-pNA (405 nm). The method was calibrated with known concentrations of pNA. To measure caspase 8-like activity, 80 μg of total protein dissolved in reaction buffer containing 10% sucrose, 10 mM dithiothreitol (DTT), 0.1% 3[(3-cholamidopropyl) dimethylammonio]-propanesulfonic acid, 25 mM HEPES (pH 7.4) was added directly into a 96 well black polysterene microplate (CLS3603, Sigma-Aldrich, USA). To initiate the reaction, 5 μL of 1 mM substrate for caspase-8 (Ac-LETD-AFC, final concentration 50 μM) was added, and the reaction mixture was incubated by 2 h at 37 °C. Caspase-like activity was measured fluorometrically with 400 nm excitation and 505 nm emission in a Biotek Cytation 3 reader (Biotek Instruments, Winooski, VT, USA). The method was calibrated with a standard curve of AFC (7-amino-4-trifluoromethyl coumarin).

### 2.13. Measurement of Caspase 3/7-Like Activity

Caspase-Glo 3/7 (Promega, Madison, WI, USA) is a homogenous chemiluminescent kit available and widely used to measure apoptosis. Compound-treated plates were prepared as described above and after incubation time, caspase-3/7 activity was measured using Caspase-Glo 3/7 following the manufacturer’s instructions. Briefly, 100 μL per well Caspase-Glo 3/7 reagent was added to the cells, and plates were agitated for 2 h in the dark at room temperature before luminescence was measured using a microplate reader (Cytation 3; BioTek US, Winooski, VT, USA).

### 2.14. Cell Metabolic Activity

Cells were seeded in 96-well plate and then subjected to the different treatments. After incubation time, the cell metabolic activity was assessed through the resazurin reduction assay [36]. The culture medium was discarded, and cells were incubated for 1 h with 80 μL of culture medium supplemented with 10 μg/mL resazurin. The appearance of resorufin, indicative of metabolic activity, was measured fluorimetrically with 570 nm excitation and 600 nm emission in a Biotek Cytation 3 reader (Biotek Instruments, Winooski, VT, USA).

### 2.15. Cell Mass

Cells were seeded in 96-well plate and then subjected to the different treatments. After incubation time, the sulforhodamine B (SRB) assay was performed for cell mass determination based on the measurement of cellular protein content [31]. Briefly, the cell culture medium was discarded, and wells rinsed with PBS 1X. Cells were fixed by adding 1% acetic acid in 100% methanol for at least 2 h at −20 °C. The fixation solution was then discarded, and the plates were dried at 37 °C. 150 µL of 0.05% SRB in 1% acetic acid solution was added and incubated at 37 °C for 1 h. The wells were then washed with 1% acetic acid in water and dried. Then, 100 μL of Tris (pH 10) was added and the plates were stirred for 15 min and optical density was measured at 540 nm in Biotek Cytation 3 reader (Biotek Instruments, Winooski, VT, USA).

### 2.16. Intracellular ATP Levels

Cells were seeded in 100 µL of culture medium, in a white opaque-bottom, 96-well plate, and then subjected to the different treatments. After incubation time, intracellular ATP levels were measured using CellTiter-Glo Luminescent Cell Viability Assay (Promega, WI, USA). Briefly, 50 µL of culture medium was removed from the wells and 50 µL of medium containing CellTiter-Glo Reagent (CellTiter-Glo Buffer + CellTiter-Glo Substrate) was added to the cells. Contents were mixed for 2 min on an orbital shaker to induce cell lysis and, after 10 min of incubation at 22 °C, the luminescence signal was monitored in a Cytation 3 reader (Biotek Instruments, Winooski, VT, USA). An ATP standard curve was also generated following the manufacturer’s instructions.

### 2.17. Computational Data Analysis

Data analysis comprised the computation of correlation matrices to summarize correlations between every pair of variables, the estimation of individual feature importance regarding how useful they are to identify the different groups of the study, and the definition of clusters to group similar samples. The correlation was computed using the Pearson coefficient, whose values belong to the interval [−1, +1]: +1 signals a total positive linear correlation, 0 identifies no linear correlation, and −1 refers to a total negative linear correlation. The individual feature importance for determining the target was estimated by calculating the mutual information gain measure, i.e., by applying a nonparametric method that approximates the decrease of entropy [37]. Non-hierarchical clustering was performed with the K-means algorithm [38], after standardization of the data. The computational analysis of the data was performed using Python 3, version 3.7.3. We relied on the Pandas [39], NumPy [40], and SciPy [41] packages to load, store and transform the data. Correlations were calculated with Pandas, whereas mutual information gain and clustering were performed with scikit-learn [42]. All data analysis figures were created with Matplotlib and Seaborn modules.

### 2.18. Statistics

Data were analyzed in GraphPad Prism 8.02 software (GraphPad Software, Inc.). Unless stated otherwise, data from multiple experiments is presented as the mean ± standard error of the mean (SEM) and statistical significance was assessed using a 2-way ANOVA with Tukey multiple comparison post-test to compare more than two groups with two independent variables (treatment and time). A 2-way ANOVA with Sidak multiple comparison post-test allowed the comparation between different time incubations in the same group. Significance was accepted with * *p* < 0.05, ** *p* < 0.01, *** *p* < 0.0005, **** *p* < 0.0001 for comparisons between treatment vs. CTL and ^#^ *p* < 0.05, ^##^ *p* < 0.01, ^###^ *p* < 0.0005, ^####^ *p* < 0.0001 for comparisons during time in the same group (24 and 6 h vs. 1 h). Significance for additional fructose effect as accepted with ^$^ *p* < 0.05, ^$$^ *p* < 0.01, ^$$$^ *p* < 0.0005.

## 3. Results

### 3.1. Supra-Physiological Concentrations of FA Increase the Accumulation of Lipid Droplets

**Figure 1:**
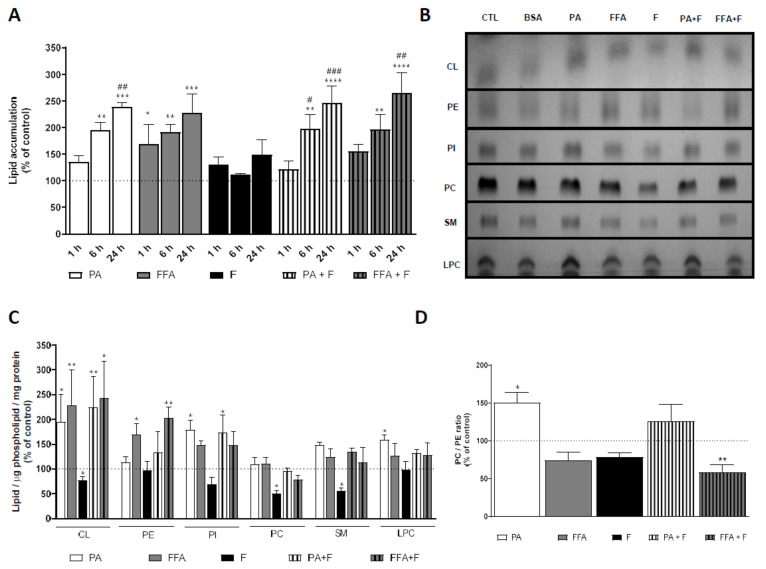
Effect of supra-physiological concentrations of FA on the accumulation of lipid and mitochondrial phospholipids content. (**A**) Lipid droplet content in HepG2 cells treated with palmitic acid (PA, 0.5 mM) or a mix of free fatty acids (FFA, 0.25 mM) in the presence or absence of fructose (F, 10 mM) for 1, 6, and 24 h. (**B**) A typical chromatogram showing the mitochondrial phospholipids profile (CL, cardiolipin; PE, phosphatidylethanolamine; PI, phosphatidylinositol; PC, phosphatidylcholine; SM, sphingomyelin; LPC, lysophosphatidylcholine) of HepG2 cells treated with PA or FFA in the presence or absence of F for 24 h. This image was inverted and contrast-optimized for visualization purposes. Quantification of the bands was performed using the original images. (**C**) Quantification of phospholipid content (CL, PE, PI, PC, SM, and LPC) in multiple experiments. (**D**) PC/PE ratios obtained in all conditions. Data are the mean ± SEM of four independent experiments, and the results normalized on the control condition (CTL = 100%, marked by a dotted line). Significance was accepted with * *p* < 0.05, ** *p* < 0.01, *** *p* < 0.0005, **** *p* < 0.0001 for comparations between treatment vs. CTL (BSA 0.01 g/mL) and ^#^ *p* < 0.05, ^##^ *p* < 0.01, ^###^ *p* < 0.0005 for comparations during time in the same group (24 and 6 h vs. 1 h).

Human hepatocarcinoma cells (HepG2) were incubated for a period of 1, 6, and 24 h with palmitic acid (PA; 0.5 mM) or with a mix of free fatty acid (FFA; 0.25 mM) [29] in the absence and presence of F (10 mM). The cytotoxic effects of each treatment were evaluated through changes in intracellular lipid content using Nile Red staining. PA and FFA treatments significantly increased intracellular neutral lipid content in a time-dependent manner (Figure 1A). Fructose by itself neither increased intracellular lipid content nor aggravated the effects of PA or FFA (Figure 1A). In agreement with results from neutral lipid droplets accumulation, the intracellular lipid content increased in the order: CTL ≈ F <<< FFA ≈ FFA + F ≈ PA ≈ PA + F. Taken together, these results demonstrate that the increase in intracellular lipid content is mainly due to the FA added, as the presence of sugar has a residual role on the different degrees of hepatic steatosis.

### 3.2. Fructose Treatment Increased HepG2 Fructokinase Protein Levels

Fructose overload can lead to a large, rapid increase in the hexose-and triose-phosphate pools, potentially increasing substrate delivery for central carbon metabolic pathways increasing the risk for the development and progression of NAFLD [43,44]. In this context, we measured the protein amount of the first enzyme responsible for fructolysis after 24 h incubation with PA, FFA, F, PA + F, and FFA + F. F treatments increased hepatic fructokinase (Ketohexokinase; KHK) protein level, mainly in F and FFA + F groups (Supplementary Figure S1).

Control liver homogenates from 16-week-old C57BL/6J mice was used as control, showing that KHK is noticeably more expressed in mouse liver than in HepG2 cell extracts, as expected (Supplementary Figure S1). These results suggest that the initial conversion of F to Fructose-1-P is likely to occur in HepG2 cells, although lipid accumulation resulting from F treatment was not observed under these experimental conditions.

### 3.3. Supra-Physiological Concentrations of fa Altered Mitochondrial Phospholipid Content

Given the higher amount of neutral lipids accumulation in cells under different treatments, we next investigated whether PA and FFA, alone or in combination with F for 24 h could alter the phospholipid composition of HepG2 mitochondrial-enriched fractions (Figure 1B). The analysis of phospholipid classes upon separation by thin-layer chromatography (TLC) showed no alterations in phosphatidylcholine (PC) content under the different PA- and FFA-treatment regimens. However, there was an increased PE content for FFA alone or in combination with F (Figure 1C). Among the most abundant phospholipids in mitochondria, cardiolipin showed an increased content for all PA- or FFA-treatments (Figure 1C). Moreover, PA and PA + F treatment resulted in a significant increase in phosphatidylinositol (PI) content, and treatment with PA alone also resulted in a significant increase in LPC (Figure 1C). Interestingly, treatment with F, by itself, significantly decreased CL, PC, and SM (Figure 1C). On the other hand, PA-treatment significantly increased the PC/PE ratio, and FFA + F treatment decreased this parameter (Figure 1D). Overall, our results demonstrate that PA treatment caused broader changes in the content of mitochondrial membrane phospholipids.

### 3.4. Supra-Physiological Concentrations of fa Time-Dependently Increase Cm-H_2_dcfda Oxidation

**Figure 2:**
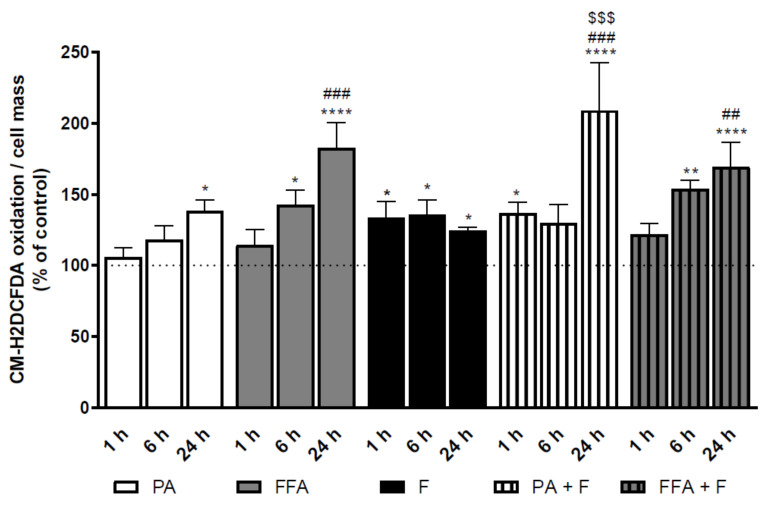
Time-dependent effect of supra-physiological concentrations of FA on the levels of CM-H_2_DCFDA-oxidizing ROS. Average cellular CM-H_2_DCFDA oxidation signal in cells treated with palmitic acid (PA, 0.5 mM) or a mix of free fatty acids (FFA, 0.25 mM) in the presence or absence of fructose (F, 10 mM) for 1, 6 and 24 h. Data are the mean ± SEM of four independent experiments, and the results normalized on the control condition (CTL = 100%, marked by a dotted line). Significance was accepted with * *p* < 0.05, ** *p* < 0.01, **** *p* < 0.0001 for comparisons between treatment vs. CTL (BSA 0.01 g/mL) and ^##^ *p* < 0.01, ^###^ *p* < 0.0005 for comparisons during time in the same group (24 and 6 h vs. 1 h). Significance for additional fructose effect as accepted with ^$$$^ *p* < 0.0005.

We next studied the effect of the different treatment groups in cellular oxidative stress by following CM-H_2_DCFDA dye. PA- and FFA, alone or in combination with F, induced a significant time-dependent increase in intracellular ROS levels (Figure 2). Fructose by itself showed increased oxidative stress in comparison with control cells for all analyzed time points, although no time-dependent effects were observed (Figure 2). Interestingly, PA- but not FFA-induced dye oxidation was further increased by F (Figure 2). Taken together, these results demonstrate that an increase in intracellular oxidative stress paralleled the increase in intracellular neutral lipid content and some phospholipids classes.

### 3.5. Supra-Physiological Concentrations of FA Altered Mitochondrial Membrane Potential (ΔΨm) and Induced Changes in Mitochondrial Morphology

In order to determine whether PA or FFA treatments alone or in combination with F affected mitochondrial morphology and ΔΨm, HepG2 cells were labeled with the fluorescent dyes Hoechst (nuclear) and TMRM (polarized mitochondria) and visualized by confocal fluorescence microscopy.

**Figure 3:**
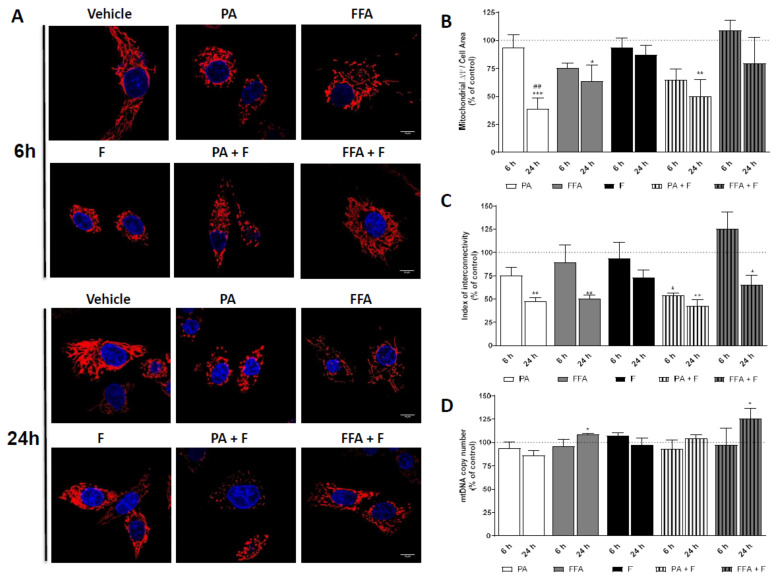
Effect of supra-physiological concentrations of FA on mitochondrial morphology and mtDNA copy number. (**A**) Typical background-corrected (COR) image of HepG2 cells stained with the fluorescent cation TMRM and Hoechst 33,342 after treatment with palmitic acid (PA, 0.5 mM) or a mix of free fatty acids (FFA, 0.25 mM) in the presence or absence of fructose (F, 10 mM) for 6 and 24 h. The TMRM and Hoechst fluorescence intensity was color-coded to red and blue, respectively. (**B**) Average mitochondrial TMRM fluorescence intensity calculated from the images. (**C**) Index of mitochondrial interconnectivity calculated from the images (**D**) mtDNA copy number in HepG2 cells treated with PA or FFA in the presence or absence F for 6 and 24 h. mtDNA copy number was based on the amplification of cytochrome B (encoded on the mitochondrial genome) and β-2-microglobulin (encoded on the nuclear genome) ratio. Data are the mean ± SEM of three independent experiments, and the results normalized on the control condition (CTL = 100%, marked by a dotted line). Significance was accepted with * *p* < 0.05, ** *p* < 0.01, *** *p* < 0.0005 for comparisons between treatment vs. CTL (BSA 0.01 g/mL) and ^##^ *p* < 0.01 for comparisons during time in the same group (24 and 6 h vs. 1 h).

PA or PA + F treatments induced time-dependent alterations in mitochondria structure, including conversion into small round-shaped structures, likely the result of mitochondria fragmentation (Figure 3A). Accordingly, a 60% decrease in ΔΨm was observed for these treatment regimens at 24 h (Figure 3A). These alterations were paralleled by the observation of warped nuclei in PA- or PA + F- treated cells in contrast with more circular-like structures observed in control cells (Figure 3A). FFA alone or combined with F also showed time-dependent mitochondrial fragmentation events, as proven by the decrease in the index of interconnectivity (Figure 3C), with no morphological differences in nuclei shape compared with control (Figure 3A). A decrease in ΔΨm was observed under those conditions, but the difference only reached statistical significance for FFA treatment (Figure 3B). Fructose alone did not affect either cell morphology or ΔΨm (Figure 3A,B). Taken together, the data suggests that FA decreased ΔΨm, which could result in or result from alterations in mitochondrial structure and integrity.

### 3.6. Supra-Physiological Concentrations of Unsaturated FA Significantly Increase mtDNA Copy Number

To understand whether mitochondrial alterations are accompanied by variations in mitochondrial DNA (mtDNA), we next studied the time-dependent impact of the different treatment groups on mtDNA copy number. No changes were observed on mtDNA copy number in cells treated PA ± F (Figure 3D). Interestingly, FFA, regardless of F, time-dependently increased mtDNA copy number (Figure 3D). Fructose by itself neither increased mtDNA copy number nor aggravated the effects observed for PA- or FFA-treatment regimens (Figure 3D). The results suggest that the FFA-treatment regimens lead to different mtDNA content, resulting in or resulting from different oxidative damage, being the later more severe.

### 3.7. Supra-Physiological Concentrations of FA Altered Level of OXPHOS Subunits

**Figure 4:**
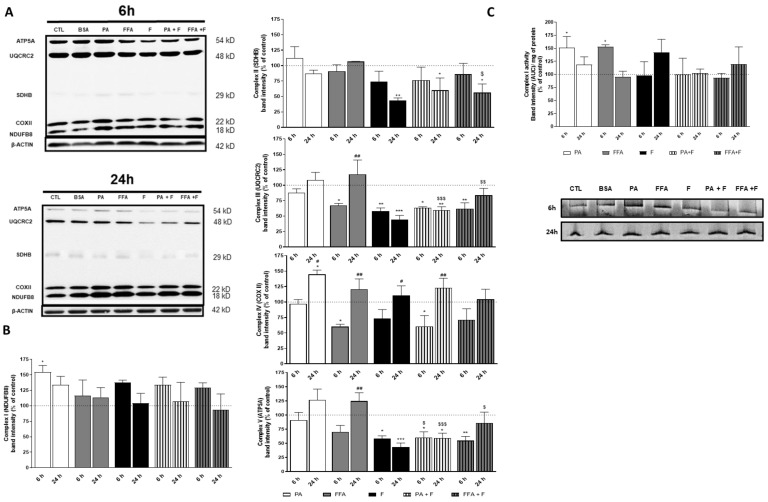
Effect of supra-physiological concentrations of FA on mitochondrial OXPHOS protein levels. (**A**) Typical Western blot result of whole cell homogenates showing the protein level of NDUFB8 (complex I), SDHB (complex II), UQCRC2 (complex III), COXII (complex IV), ATP5A (complex V) subunits, and β-actin (cytosolic marker) in cells treated with palmitic acid (PA, 0.5 mM) or a mix of free fatty acids (FFA, 0.25 mM) in the presence or absence of fructose (F, 10 mM) for 6 and 24 h. This blot was inverted and contrast-optimized for visualization purposes. Quantification of the bands was performed using the original blots. (**B**) Quantification of OXPHOS proteins levels in multiple experiments normalized to β-actin levels and for the control group (100% marked by a dotted line). (**C**) Typical BN-PAGE in-gel activity result of mitochondrial-enriched fraction homogenates depicting the protein activity of mitochondrial complex I (NADH:ubiquinone oxidoreductase) in cells treated with palmitic acid (PA; 0.5 mM) or a mix of free fatty acids (FFA; 0.25 mM) in the presence or absence of fructose (F; 10 mM) for 6 and 24h. This image was inverted and contrast-optimized for visualization purposes. Quantification of the bands was performed using the original images. Data are the mean ± SEM of four independent experiments, and the results normalized on the control condition (CTL = 100%, marked by a dotted line). Significance was accepted with * *p* < 0.05, ** *p* <0.01, *** *p* < 0.0005 for comparisons between treatment vs. CTL (BSA 0.01 g/mL) and ^#^ *p* < 0.05, ^##^ *p* < 0.01 for comparisons during time in the same group (24 and 6 h vs. 1 h). Significance for additional fructose effect as accepted with ^$^ *p* < 0.05, ^$$^ *p* < 0.01, ^$$$^ *p* < 0.0005.

In order to determine whether fatty acids overload is implicated with mitochondrial defects in a time-dependent manner, we semi-quantified protein levels for OXPHOS Complex I (NDUFB8), complex II (SDBH), complex III (UQCRC2), complex IV (COXII), and complex V (ATP5A) subunits (Figure 4A). PA or PA + F increased the level of complex I NDUFB8 subunit by ~50% at 6 h followed by a decrease to control levels at the 24 h time point (Figure 4B). Although no alterations were found for complex II SDBH subunit at 6 h for any of the treatments, the presence of F in combination with fatty acids (PA or FFA) decreased this subunit at 24 h (Figure 4B). The level of complex III UQCRC2 subunit was decreased at 6 h in PA and FFA treatments in the presence of F, while in the absence of this sugar, only the FFA treatment resulted in the same effect. Similarly, F also decreased the protein levels of complex III UQCRC2 subunit.

Interestingly, this effect was also observed at 24 h for F and PA + F treatments, while the levels of this protein recovered to control levels after FFA + F treatment (Figure 4B). Except for PA treatment, all the remaining treatments significantly reduced the protein levels of complex IV COXII subunit at 6 h. Interestingly, the levels of COXII subunit were equal in all experimental conditions (Figure 4B). The level of ATP5A subunit was decreased in PA, and FFA treated cells in the presence of F at 6 h, while in the absence of F, no alterations were observed for the same time point. Similarly, F also decreased the level of this subunit of complex V. Interestingly, in F and PA + F treatment, this effect was also observed at 24 h, while in FFA + F treatment, the levels of complex V subunit recovered to control level at the same time-point (Figure 4B).

### 3.8. Supra-Physiological Concentrations of FA Altered Native Mitochondrial Electron Transport Complex I Activity

Altered mitochondrial respiration resulting from impairment of OXPHOS complexes activity has been described as a primary “hit” of NAFLD progression to NASH [45,46]. To investigate the contribution of these abnormalities to the final dysfunction outcome, we measured the in-gel activity of the major OXPHOS complex (Complex I) using BN-PAGE electrophoresis at 6 and 24 h. PA- and FFA-treatment showed increased CI activity at 6 h, followed by a decrease to control values at 24 h (Figure 4C). Fructose-related treatments did not affect complex I activity (Figure 4C).

These results suggest an early increase of CI activity to overcome the high accumulation of FA, with a later decrease possibly due to the excessive fatty acids β-oxidation and ROS production.

### 3.9. Supra-Physiological Concentrations of FA Time-Dependently Decrease Oxygen Consumption Rates (OCR) and Increased Extracellular Acidification Rates (ECAR)

**Figure 5:**
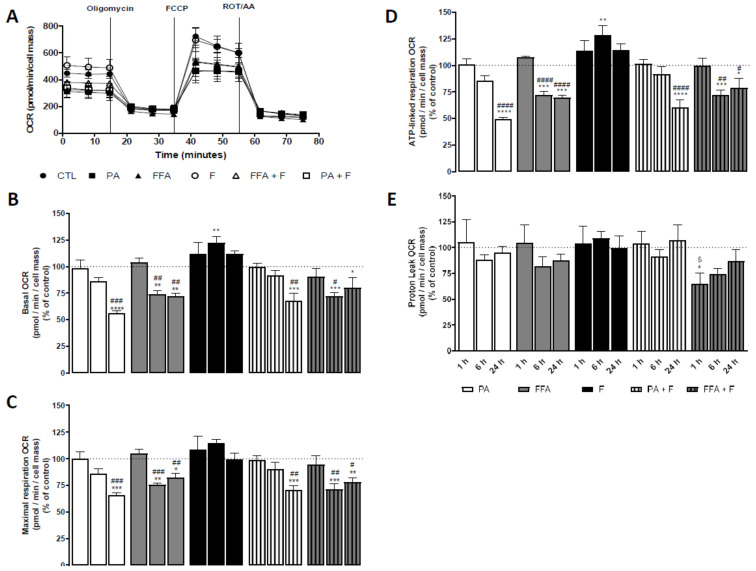
Time-dependent effect of supra-physiological concentration of FA on mitochondrial oxygen consumption. (**A**) Typical representation of oxygen consumption rate (OCR) measurement in HepG2 cells treated with palmitic acid (PA, 0.5 mM) or a mix of free fatty acids (FFA, 0.25 mM) in the presence or absence of fructose (F, 10 mM) for 1, 6 and 24 h. Several respiratory parameters were evaluated: (**B**) cellular basal respiration; (**C**) maximal respiration; (**D**) ATP production-linked respiration; and (**E**) proton leak. Data are mean ± SEM (expressed as pmol O_2_/min/cell mass) of four independent experiments, and the results normalized on the control condition (CT = 100%, marked by a dotted line. The data obtained for the different treatments was compared with the control group. Significance was accepted with * *p* < 0.05, ** *p* < 0.01, *** *p* < 0.0005, **** *p* < 0.0001 for comparisons between treatment vs. CTL (BSA 0.01 g/mL) and ^#^ *p* < 0.05, ^##^ *p* < 0.01, ^###^ *p* < 0.0005, ^####^ *p* < 0.0001 for comparisons during time in the same group (24 and 6 h vs. 1 h). Significance for additional fructose effect as accepted with ^$^ *p* < 0.05. Legend: FCCP—carbonyl cyanide 4-(trifluoromethoxy)phenylhydrazone, ROT—rotenone, AA—antimycin A.

In order to determine whether fatty acid overload, in the absence or presence of F, affects mitochondrial function, the oxygen consumption rate (OCR) of HepG2 cells was measured by using the Seahorse XF-96 Extracellular Flux Analyzer (Figure 5A).

PA- and FFA-treatments significantly decreased basal (Figure 5B), ATP-linked (Figure 5D), and maximal (Figure 5C) OCR respiration in a time-dependent manner, while only FFA + F, for the 6 h time-point, affected proton leak respiration (Figure 5). Fructose by itself neither altered mitochondrial OCR nor aggravated the effects observed for PA- or FFA-treatment regimens (Figure 5). The extracellular acidification rate (ECAR), primarily resultant from extrusion of protons to the surrounding medium when lactate is produced, was increased in a time-dependent manner for F-treated cells (Supplementary Figure S2A). However, no alterations were observed in PA- or FFA-treated cells (Supplementary Figure S2A). The average data are plotted in Figure 5B and Supplementary Figure S2A. At the data points tested, cells subjected to PA treatment were shifted for a more quiescent status, while F-treated cells shifted for a more energetic status compared to control data points (Supplementary Figure S2B).

### 3.10. Supra-Physiological Concentrations of FA Induced Caspases Activation in HepG2 Cells Which Follows ROS and Mitochondrial Dysfunction

**Figure 6:**
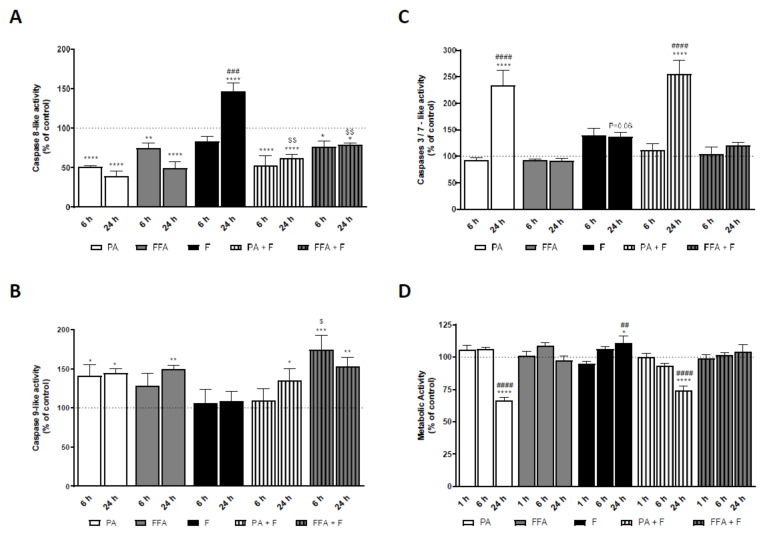
Time-dependent effect of fatty acid excess on caspase activity and cell viability. (**A**) Caspase 8-like activity in HepG2 cells treated with palmitic acid (PA, 0.5 mM) or a mix of free fatty acids (FFA, 0.25 mM) in the presence or absence of fructose (F, 10 mM) for 6 and 24 h. (**B**) Same as panel A but now for caspase 9-like activity. (**C**) Same as panel A but now for caspase 3/7. (**D**) Cell viability of HepG2 cells cultured in the presence of palmitic acid (PA, 0.5 mM) or a mix of free fatty acids (FFA, 0.25 mM) in the presence or absence of fructose (F, 10 mM) for 6 and 24 h. Data are the mean ± SEM of four independent experiments, and the results normalized on the control condition (CT = 100%, marked by a dotted line). Significance was accepted with * *p* < 0.05, ** *p* < 0.01, *** *p* < 0.0005, **** *p* < 0.0001 for comparisons between treatment vs. CTL (BSA 0.01 g/mL) and ^##^ *p* < 0.01, ^###^ *p* < 0.0005, ^####^*p* < 0.0001 for comparisons during time in the same group (24 and 6 h vs. 1 h). Significance for additional fructose effect as accepted with ^$^ *p* < 0.05, ^$$^ *p* < 0.01.

In order to determine whether mitochondrial dysfunction resulting from fatty acid treatment precedes caspase-dependent apoptotic pathways, caspases 8, 9, and 3/7- like activities assays were performed. PA and FFA treatments, in the presence or absence of F significantly decreased caspase 8-like activity at 6 and 24 h. On the other hand, F alone increased caspase 8-like activity at 24 h time-point (Figure 6A). Regarding caspase 9-like activity, FFA alone or in combination with F significantly increased its value at 24 h time-point. A slight increase was observed in PA treatments alone or in combination at 24 h, but only PA-treated cells showed an increased caspase 9-like activity at 6 h (Figure 6B). On the other hand, PA alone or in combination with F, significantly increased caspases-3/7 activities in a time-dependent manner (Figure 6C), while FFA treatment had no effects on caspase 3/7 activity (Figure 6C).

Together, these results suggest that fatty acids induced an early increase in the mitochondrial-independent apoptotic pathway, as observed for the decrease in caspase 8-like activity. Moreover, the activation of mitochondrial-dependent caspase activation appears to be relevant in PA and FFA treatments. The data reinforce the idea that FFA- and PA-treatments lead to different apoptotic outcomes, being the latter more severe, as observed for the significant increase in caspase 3/7 activities.

### 3.11. Supra-Physiological Concentrations of Saturated FA Time-Dependently Decrease Intracellular ATP Levels

Considering all observed alterations in the mitochondrial activity, we measured cellular ATP levels in all treatment groups. PA-treatment (+/− F) significantly decreased ATP levels in a time-dependent manner, while no changes were observed in the case of FFA (Figure S2C). Interestingly, F by itself promoted an initial decrease in intracellular ATP levels, which were restored to control levels after 24 h (Figure S2C), a pattern that was also observed for FFA + F treatment (Figure S2C). These results suggest that the increase in intracellular lipid droplets did not significantly alter intracellular ATP levels. Moreover, the absence of a dramatic drop in intracellular ATP levels suggests that FA-induced cell damage may involve apoptotic and not necrotic cell death mechanisms.

### 3.12. Supra-Physiological Concentrations of FA Time-Dependently Decrease Cell Metabolic Activity and Mass of Human Hepatocarcinoma Cells

Final endpoints for lipotoxicity were evaluated by measuring cell metabolic activity changes (NADH/NADPH dehydrogenase activity) and mass using the resazurin reduction and sulforhodamine B (SRB) assays, respectively. PA regimens reduced HepG2 metabolic activity (Figure 6D) and mass (Figure S2D) in a time-dependent manner, while FFA treatment had no effects on cell metabolic activity (Figure 6D) or mass (Supplementary Figure S2D).

Fructose by itself increased metabolic activity at 24 h (Figure 6D), whereas no alterations were observed in cell mass (Supplementary Figure S2D). Moreover, F did not aggravate any of the effects caused by PA- or FFA-treatments alone (Figure 6D and Figure S2d). These results demonstrate that different fatty acids treatment regimens (FFA or PA) induced different changes in cell viability and mass, being the later treatment more severe, as observed for the significant decrease in cell viability.

### 3.13. Exploratory Data Analysis Separated PA and FFA Regimens By Identifying a Subset Of Critical Mitochondrial Markers

In the present work, we applied exploratory and unsupervised computational data analysis techniques to gain insight into the relations between the experimental data considered for this study and identify a minimal subset of critical mitochondrial markers that can be used to investigate hallmarks of in vitro NAFLD models. The small number of samples prevents a complete and robust statistical analysis of the results, but it nevertheless allows for identifying relevant hidden patterns and trends.

**Figure 7:**
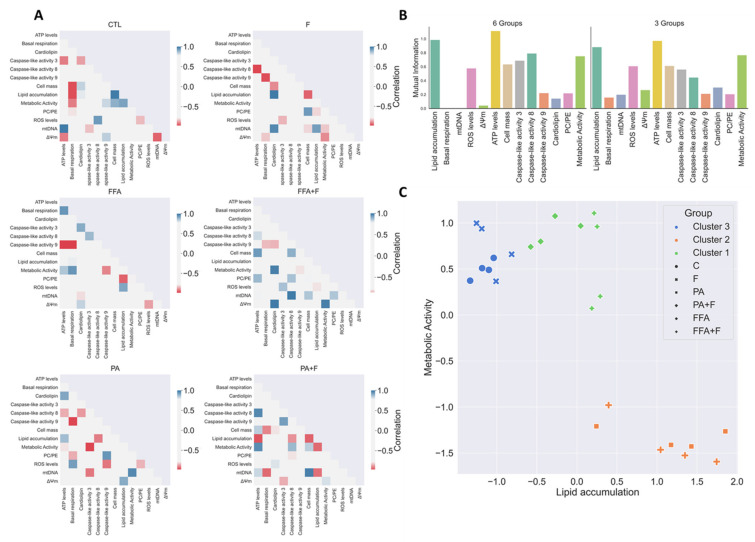
Computational data analysis of all experimental endpoint measures analyzed in different lipotoxicity models on human hepatocytes. (**A**) Correlation matrices of HepG2 cells treated with palmitic acid (PA, 0.5 mM) or a mix of free fatty acids (FFA, 0.25 mM) in the presence or absence of fructose (F, 10 mM). (**B**) Mutual information gain of each individual experimental endpoint (24 h), regarding the existence of 3 or 6 experimental groups. (**C**) K-means clustering results, for k = 3, using a subset of selected experimental endpoints.

The 6 matrices from Figure 7A present the pairwise correlations for all experimental endpoint measures considered in the study. To enhance clarity and focus the analysis on the most relevant correlations, only absolute values above 0.7 are shown. We present results separately for the PA and FFA treatments, with and without F. For completeness, we also present results obtained in a media without any of the treatments. The analysis of the results unveils the existence of different correlation profiles regarding PA and FFA treatment groups. While the PA treatment shows a strong positive correlation between PC/PE and caspase 9 activation, the same was not observed in the FFA regimen. Interestingly, a clear negative correlation is observed when comparing ATP levels and basal respiration to caspase 9 activation in FFA treatment. Moreover, metabolic activity correlates negatively with caspase 9 activation, suggesting an important role of these mitochondrial markers in downstream cell death events for this treatment.

Individual mutual information takes every experimental endpoint separately and estimates how relevant this measure is to differentiate targets. It is an appropriate step, e.g., in the process of building decision trees [37] and, when considered in the early stages of an exploratory data analysis, it identifies a subset of features that might be particularly relevant to separate experimental samples with different treatments. In this step, we consider two alternative target definitions: on the first stage, we consider 6 targets {CTL, F, PA, PA + F, FFA, FFA + F}; on a second approach, we aggregate the experimental samples of the original targets two-by-two, ending up with the following groups: {{CTL,F},{ PA, PA + F}, {FFA, FFA + F}}. Charts presented in Figure 7B display the individual mutual information for the two scenarios. A brief inspection of the results reveals that the pattern is similar for both situations: ATP levels, lipid accumulation, metabolic activity, ROS levels, cell mass and caspase 3 and 8 are the most relevant features to individually estimate the target, both in the detailed and aggregated scenarios. There are some slight variations, but no single experimental endpoint exhibits a clear different behavior when moving from one situation to the other.

To complete the computational analysis, we applied a non-supervised learning method to verify if it is possible to separate the samples considering just the information provided by a limited subset of experimental endpoints. Building on the analysis of the mutual information, we selected the following endpoints: {Lipid accumulation, ATP levels, Caspase 3, Caspase 9, mtDNA, Metabolic Activity}. All these features exhibit a comparatively high discriminative power of the targets, except for mtDNA. Nevertheless, we verified that this last endpoint was essential for good separation between groups. After standardization, the k-means clustering algorithm was applied to the selected data. When the parameter specifying the number of clusters to form is set to 3, the result obtained is displayed in Figure 7C. Three well-formed clusters, exhibiting good cohesion and separation, are reported by k-means. The clusters are perfectly homogeneous when considering the aggregated three experimental groups previously defined, confirming that the selected subset of features can separate targets. We did some additional experiments and verified that it is impossible to separate the six original groups considering any subset of the experimental endpoints. One result from the computational analysis is that the presence or absence of F does not interfere with any separation of the different groups studied, at least in the cell lines used. Indeed, the output of computational analysis strongly correlates with the biological outcomes observed, reinforcing the crucial importance of mitochondria in apoptotic pathways in fatty acid overload scenarios.

## 4. Discussion

Supra-physiological accumulation of FA and/or sugars overload in hepatic cells lead to a significant increase in lipid accumulation in the cytoplasm, which can be toxic after a concentration threshold is reached [27]. Here, we investigated time-dependent cellular effects of different in vitro FA overload strategies in the presence or absence of F to understand the chronological events leading to mitochondrial dysfunction upon supra-physiological FA exposure. Exploratory and unsupervised computational approaches identified a minimal subset of critical mitochondrial markers that can be used to predict cell death in NAFLD models on human hepatocytes to create a model for high throughput screening of possible therapeutic agents, with a particular focus on measuring mitochondrial function.

Lipotoxicity driven by high concentrations of lipids and/or carbohydrates exposure has been reported in several in vitro studies [3,4,47,48,49]. However, different cellular outcomes were attained depending on lipid composition and/or the number of double bonds, which suggest different cellular responses depending on FA composition and time of exposure. We used a well-established human hepatoma cell model (HepG2) owning to the fact that these cells contain major lipid metabolizing enzymes such as triglyceride lipase or 3-hydroxy-3-methyl-glutaryl-coenzyme A reductase (HMG-CoA reductase) [50], which validate that cell line as a proper model for steatotic phenotype studies. To study FA overload, we exposed HepG2 cells to PA, which is the most abundant saturated FFA in mammals and a mixture of FFA (39% C16:0; 5% C18:0; 50% C18:1; 4% C18:2; 2% C20:4), in order to mimic FFA composition in a liver of 24 weeks Western diet-fed mice [28,29]. The novelty of this work was that we performed initial time-dependent experiments on lipids droplets accumulation and demonstrated that both PA and FFA induced a progressive steatotic phenotype. Although the FFA mixture has been used at half the PA concentration, an identical steatotic phenotype was measured. This result follows previous studies demonstrating that FA structure and the cellular availability of FA affect lipid droplets accumulation [51]. The addition of F did not result in the accumulation of lipid droplets by itself and did not aggravate the degree of hepatic steatosis when combined with FA.

Interestingly, an increased protein amount of hepatic fructokinase was observed in the F group, suggesting that fructolysis may be stimulated by F treatment in this cell model. The small decrease in ATP levels observed at 6 h hours for F treatment reinforces the idea of an efficient conversion of F to F-1-P. This result is in accordance with previous studies demonstrating that the initial decreased ATP levels for treatments involving F can be due to the rapid conversion of F to F-1-P and later to F-1,6-bis P in liver cells, which are both ATP-dependent processes [52]. However, in HepG2 cells, the rate of this process seems to be lower when compared with mouse liver homogenates, as observed by the higher amount of KHK, probably explaining the lack of lipid droplets accumulation in F treatments. Although metabolic effects of F were well-documented in these experimental conditions, more extended incubation periods should be addressed to reflect TG accumulation in HepG2 cells.

Cellular (phospho)lipid homeostasis is important, particularly for mitochondrial activity, as it plays an important role in coordinating the synthesis of some key membrane phospholipids [53]. The major constituting mitochondrial membrane phospholipids are phosphatidylcholine (PC) (41%) and phosphatidylethanolamine (PE) (32%) [54]. Both PA and FFA treatments showed to induce alterations in phospholipids content with a crucial role in maintaining the structure, integrity, and bioenergetics of mitochondria [55]. On the other hand, F-treated cells showed decreased levels of most of the phospholipids. Phosphatidylinositol (PI) was increased in PA and PA + F treatments, although no difference was observed with FFA (±F). Increased PI and phosphoinositides were reported and associated with vacuolar membrane formation and endocytosis [56]. PC/PE ratio alterations may modulate membrane plasticity and bending rigidity with impact on the conformation dynamics of protein transmembrane domains and regulate the appearance of lipid packing defects influencing fusion phenomena and binding and activity of peripheral membrane properties [57]. PE is highly enriched in mitochondrial inner membranes (~ 40% of total phospholipids) compared to other organelle membranes (15–25% of total phospholipids). In our work, PE was significantly increased on FFA regimens (but not in PA treatments) with no significant alterations for PC, which results in lower PC/PE ratios for FFA treatments, mainly FFA + F. This result, together with higher mtDNA content for the same conditions could indeed mean an increase in mitochondrial mass as an adaptative response to FFA overload. A recent study in hepatocytes from mice showed that lower ratios of PC/PE, a consequence of PE increase, stimulate mitochondrial respiration and activities of proteins of the electron transport chain [58].

Moreover, lower hepatic PC/PE ratios were reported in simple steatosis (SS) patients [59]. In contrast, we observed a decrease in mitochondrial respiration, which may indicate that an increase in PE content imposes curvature stress on the membranes that result not as closely packed as those formed by PC [60], creating lipid-packing defects with deleterious consequences in ΔΨm and O_2_ consumption. Interestingly, an increase in mitochondrial PC/PE molar ratio in Chinese hamster ovary cells leads to an impairment in cell survival and growth. In addition, oxygen consumption, cellular ATP levels, and the rate of ATP production were markedly reduced, consistent with defects in OXPHOS complexes [61]. Furthermore, a rise in PC/PE ratio has also been found to induce ER stress, leading, in this case, to the unfolding protein response (UPR) through disrupted calcium homoeostasis in leptin-deficient obese mice [62]. PA regimens induced a slight increase in PC/PE ratios, as well as a decrease in ATP and OXPHOS complexes, followed by cell death. This result is following previous studies demonstrating that FA overload led to changes in mitochondrial membrane phospholipids profile, possibly with deleterious effects on cellular ROS production and mitochondria function [63].

In our work, supra-physiological levels of FA, in the presence or absence of F, time-dependently increased intracellular ROS levels [3]. In fact, increased mitochondrial β-oxidation of FA, in which FA are repeatedly cleaved to produce acetyl-CoAs that feed the Krebs cycle and produce reducing equivalents for oxidative phosphorylation [27], is an important source of ROS in NASH [64]. Different levels of steatosis could correlate with stages of mitochondrial health and vice versa. Moreover, altered phospholipids content paralleled by a time-dependent increase in ROS levels supports the idea that lipid peroxidation and membrane remodeling could be related. Our work also showed that PA or PA + F treatments resulted in mitochondrial network fragmentation and decreased mitochondrial membrane potential (ΔΨm) The data suggests that the FFA-treatment regimens and PA-treatment led to different oxidative damage status, being the later more severe, and mitochondrial dysfunction, probably due to alterations in mitochondrial structure and integrity. Mitochondria are highly dynamic organelles with constant changes in shape, size, and localization around the cell [65]. Mitochondrial shapes vary from small sphere-shaped and oval to extremely interconnected filamentous networks [66]. Altered ΔΨm followed by morphological deformation of mitochondria has been reported in NAFLD, suggesting the involvement of mitochondrial dynamics in the pathogenic progression [67]. This result follows previous studies demonstrating that in vitro incubation of cells with saturated fatty acids induces mitochondrial dysfunction, playing a crucial role in NAFLD progression [68].

Mitochondrial dysfunction is commonly associated to mitochondrial fragmentation and loss of mitochondrial DNA (mtDNA) integrity [69]. FFA, but not PA, both in the absence or presence of F, time-dependently increased mtDNA copy number. This result follows previous studies demonstrating that polyunsaturated fatty acids increase mtDNA copy number in human skeletal muscle cells and mouse muscle myoblasts (C2C12) [70,71], while supra-physiological saturated fatty acid overload treatment reduced mtDNA copy number in C2C12 cells [72]. Although the initial response to high FA import would be an adaptative response to increasing the number of copies of mtDNA [73] and consequently mitochondrial biogenesis, long-term oxidative stress leads to the depletion of mtDNA alongside mitochondrial dysfunction [74]. The data reinforce the idea previously described in the literature that polyunsaturated fatty acids can increase mtDNA copy number as an adaptative response to counteract the initial oxidative stress-induced damage in HepG2 cells [70]. The increase of cardiolipin levels for different treatments also corroborates a possible adaptation mediated by mitochondrial biogenesis due to CL role in maintaining inner membrane architecture and osmotic stability and the ability to assemble the respiratory supercomplexes [61]. Several studies in rats showed increased levels of CL in the liver as a trigger to maintain or boost mitochondrial function in response to the excessive energy substrate availability [75,76]. However, excess cardiolipin has also been shown to have harmful effects on mitochondria, mainly to its highly susceptible to peroxidation driven by the proximity to respiratory chain proteins, which are the main ROS generators [77].

Mitochondrial dysfunction is commonly associated with mitochondrial fragmentation and alteration of the oxidative phosphorylation process [69]. A defective mitochondrial OXPHOS has been linked to the deleterious effects of FA accumulation and the generation of oxidative stress on hepatocytes during NAFLD [68]. The present results suggested an early increase of complex I NDUFB8 subunit possibly to overcome the excess supply of fatty acids deposition on the cell and to compensate for the decrease of OXPHOS complexes III (UQCR2), IV (COX2), and V (ATP5A) subunits, which is progressively lost with time. The data also suggests a fructose-induced decrease in the level of several OXPHOS subunits, which could be explained by the decrease in phospholipid content [78].

Excess FA critically induces ROS formation, resulting in lipotoxicity associated with ER stress, calcium dysregulation, mitochondrial dysfunction, and bioenergetics failure [79]. PA- and FFA-treatments significantly decreased basal, ATP-linked, and maximal OCR respiration in a time-dependent manner, while no changes were observed on proton leak respiration. Fructose by itself neither decreased mitochondrial function nor aggravated the effects observed for PA- or FFA-treatment regimens. The extracellular acidification rate (ECAR) increased for all treatments involving F, in a time-dependent manner, although no alterations were observed in PA- or FFA- treated cells. Consequently, PA-treated cells’ metabolic profile shifted for a more quiescent status, while F-treated cells shifted for a more energetic status compared to control data points. Taken together, these results demonstrate that the increase in intracellular lipid droplets, paralleled by an increase in intracellular ROS levels, led to mitochondrial impairments and, consequently, failure of mitochondrial bioenergetics. This result agrees with previous studies demonstrating that different FA (PA or FFA) overload progressively led to the failure of mitochondrial bioenergetics [80,81], while sugars (e.g., F) may potentiate the glycolytic machinery [82].

Excessive FA accumulation in the liver can trigger oxidative stress, due mainly to early mitochondrial adaptation and further mitochondrial dysfunction, ending with upregulating hepatocyte apoptosis via oxidative stress-mediated mechanisms [83]. So far, studies indicate that there are two main apoptotic pathways: the extrinsic or death receptor pathway and the intrinsic or mitochondrial pathway, both resulting from caspase 3 cleavage and dismantling of intracellular components [84].

Our results suggest that FA induced an intrinsic or mitochondrial apoptotic pathway as observed to decrease caspase 8-like activity and a time-dependent increase in caspase 9. Indeed, caspase-9 is required for mitochondrial morphological changes and ROS production by cleaving and activating Bid into tBid [85]. Moreover, FFA-treated cells showed a later onset in caspase activation when compared with PA-regimens. The data reinforce the idea described above that FFA treatment regimens and PA-treatment lead to different oxidative damage status, being the latter more severe, as observed for the significant increase in caspase 3/7-like activities. F-treated cells showed increased caspase 8-like activity at 24 h with no changes in caspase 9 activation, which suggests that fructose could induce apoptosis by activating the extrinsic pathway. When caspase-8 is activated, the execution phase of apoptosis is triggered in this pathway [86]. Furthermore, the presence of fructose in FA treatments increases caspase 8 activity compared with FA alone, without increasing caspase 3/7-like activities or aggravating cell viability.

This result is in accordance with previous studies demonstrating the involvement of caspase-dependent cell death in the lipotoxicity associated with fatty acid overload treatment [87] and that different FA and their cellular availability differentially affected cell’s response [4]. Moreover, a link between changes in other mitochondrial lipids and apoptosis events has been described [56]. Concentrations of lysolipids, or ceramides, above their critical micellar concentration can be cytotoxic, as they favor the permeabilization of the outer mitochondrial membrane to release apoptogenic factors into the cytoplasm, acting like detergents [88,89,90,91]. In our study, PA-treatment increased LPC levels, corroborating similar results observed in Huh-7 cells [20] and human plasma [92].

Intracellular ATP levels is an in vitro and in vivo determinant parameter of the cell’s decision to die by apoptosis or necroptosis. PA-treatment regimens (in the absence or presence of F) time-dependently significantly decreased ATP levels, while no changes were observed on FFA treatment regimen. This suggests that the increase of intracellular in intracellular lipid droplet did not significantly alter intracellular ATP levels, probably because in this treatment, cells can still supply ATP demands through glycolysis. Moreover, the absence of a dramatic drop in intracellular ATP levels suggests that FA-induced cell damage triggers apoptotic and not necrotic cell death mechanisms [93], although we did not measure necrosis markers in this work.

Activation of apoptotic caspases results in inactivation or activation of substrates, and the generation of a cascade of signaling events permitting the controlled demolition of cellular components [94]. Hereupon, PA treatment time-dependently reduced HepG2 metabolic activity and mass, while FFA treatment had no effects on cell metabolic activity or mass. Fructose by itself neither reduced cellular metabolic activity and mass nor aggravated the effects observed for PA- or FFA-treatment. The FFA regimens displayed similar features regarding PA treatments, such as decreased basal and ATP-linked respiration and ΔΨm reduction and increased ROS levels and CL content, but the energetic state of the cell was unaltered with no evidence for caspases 3/7 activation. These data demonstrate that different FA overload (FFA or PA) induced different changes on cell metabolism and death, being the latter more severe due to hepatic metabolism driven by PA, as observed for the significant decrease in cell viability. This result is in accordance with previous studies demonstrating that unsaturated FA showed protective effects against PA-induced toxicity [95,96,97].

## 5. Conclusions

**Figure 8:**
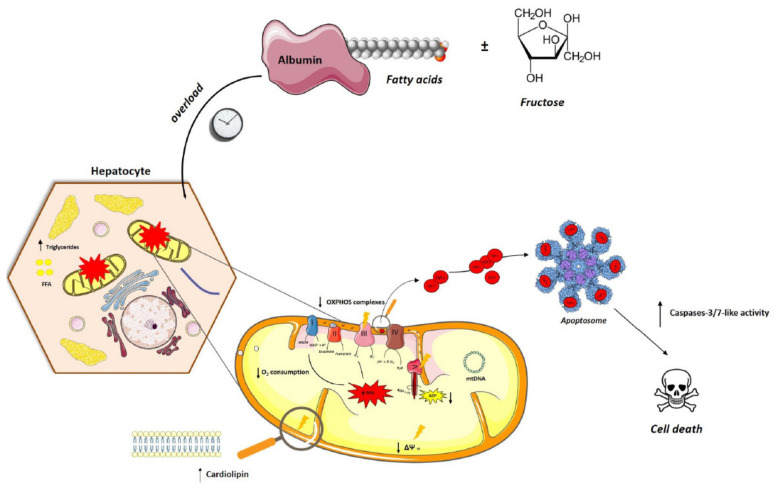
Proposed mechanism for fatty acid overload and mitochondrial dysfunction on human hepatocytes. FA overload are toxic and trigger a time-dependent caspase apoptotic cell death. The progressive increase in neutral lipids content and phospholipid modifications in mitochondria, followed by a large increase in ROS levels compromise mitochondrial function (decrease in ΔΨm, O_2_ consumption, and OXPHOS protein levels). Mitochondrial impairment can potentiate even more ROS production. ROS itself, or in conjugation with mitochondrial dysfunction, can lead to activation of caspase-dependent apoptotic cell death pathways. Thus, fatty acid overload led to a time-dependent decrease in cell viability.

In summary, we suggest a mechanism of fatty acid overload with a strong association between mitochondria dysfunction and cell death in the HepG2 in vitro model (Figure 8). Our results confirmed that both FA treatments induce a time-dependent mitochondrial apoptotic pathway, with the deleterious phenotype observed in PA regimens. In agreement, a more comprehensive understanding of how mitochondria chronologically behave in different FA ± fructose incubations in our in vitro model allowed obtaining a feasible model in which mitochondria dysfunction increased in the order: CTL < F < FFA + F ≤ FFA < PA ≤ PA + F.

The unsupervised learning algorithms used here created homogeneous and cohesive clusters, with a clear separation between PA and FFA treated samples, identifying a minimal subset of critical mitochondrial experimental endpoints that predict cell dysfunction and death in NAFLD or for high throughput screening of possible therapeutic agents.

## Data Availability

Data is available from the authors upon reasonable request.

## Supplementary Materials

The following are available online at https://www.mdpi.com/article/10.3390/nu13051723/s1, Figure S1: Effect of supra-physiological concentration of fructose on hepatic fructokinase protein level; Figure S2: Time-dependent effects of fatty acid excess on extracellular acidification, ATP levels and cell mass.
