# Supplementary material for: Exploratory Data Analysis of Cell and Mitochondrial High-Fat, High-Sugar Toxicity on Human HepG2 Cells
Source: Nutrients. 2021 May 19;13(5):1723. doi: 10.3390/nu13051723 (PMC8161147; doi:10.3390/nu13051723)
Supplement: Supplementary file 1 [file nutrients-13-01723-s001.zip › nutrients-1200498-supplementary.pdf]

Supplementary material

Animals and dietary regimen.

Four-week-old male C57BL/6J mice were purchased from SAS (Saint-Germain-Nuelles, France). The mice were fed a standard chow (CHOW) *ad libitum* starting at the age of seven weeks. The standard chow diet derives 9% of its energy from fat, 58% from carbohydrates and 33% from protein and comprises 3% fat and 5% sucrose. The CHOW (V1534) diet was purchased from Ssniff (Soest, Germany). All mice were housed in laboratory cages at 21-23 °C with 50-60% humidity (10-15 exchanges per h) with tap water and diet provided *ad libitum* for sixteen weeks. For tissue collection, the mice were anesthetized with isoflurane and further sacrificed by cervical dislocation.

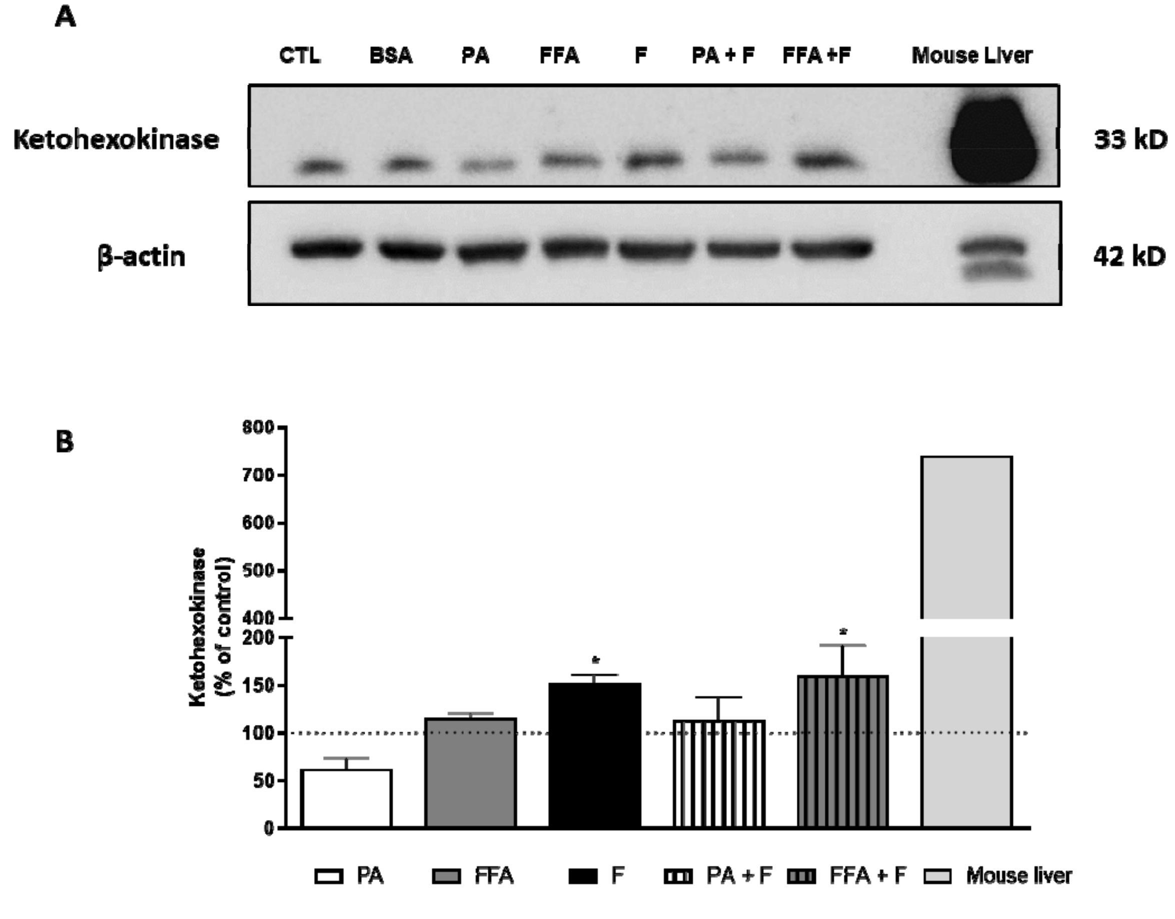

Fig. S1: Effect of supra-physiological concentration of fructose on hepatic fructokinase protein level. (**a**) Typical Western blot result of whole cell homogenates showing the protein level of Ketohexokinase (KHK) in cells treated with palmitic acid (PA, 0.5 mM) or a mix of free fatty acids (FFA, 0.25 mM) in the presence or absence of F (F, 10 mM) for 24 h. Mouse liver homogenate (30 µg) was used as positive control, as KHK is highly expressed in liver. This blot was inverted and contrast-optimized for visualization purposes. Quantification of the bands was performed using the original blots. (**b**) Quantification of KHK protein levels under the different treatment conditions normalized to β-actin levels and for the control group (100% marked by a dotted line). Data are the mean ± SEM of four independent experiments, and the

results normalized on the control condition (CT = 100 %, marked by a dotted line). Significance was accepted with *P < 0.05 for comparisons between treatment vs CTL or 0.1 % BSA.

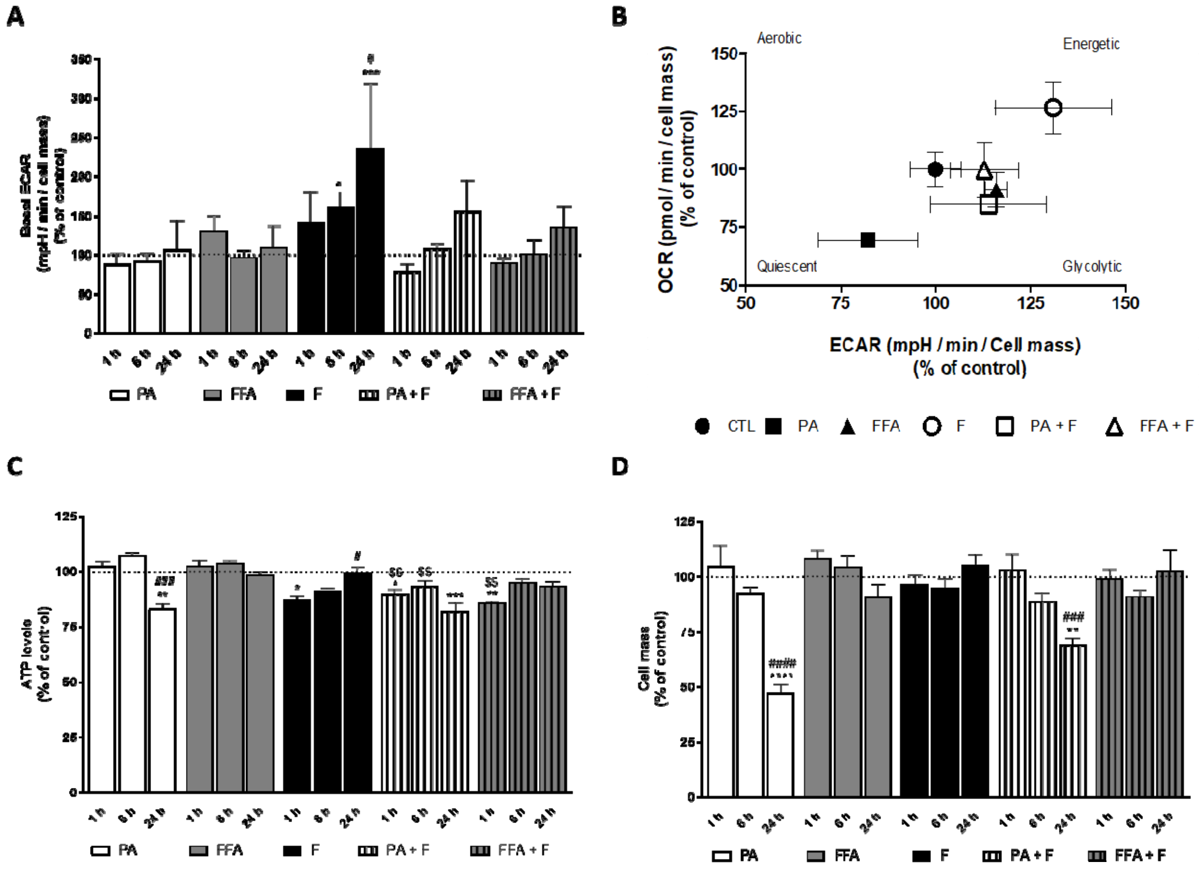

Fig. S2: Time-dependent effects of fatty acid excess on extracellular acidification, ATP levels and cell mass. (a) Basal extracellular acidification rate of HepG2 cells treated with palmitic acid (PA, 0.5 mM) or a mix of free fatty acids (FFA, 0.25 mM) in the presence or absence of fructose (F, 10 mM) for 1, 6 and 24 h. (**b**) The average mitochondrial basal OCR was plotted against the average basal ECAR (data points taken from **Fig. 5b and S2a**) in cells submitted to the treatments described above. (**c**) Same as panel A but now for cell mass density. Data are the mean ± SEM of four independent experiments, and the results normalized on the control condition (CT = 100 %, marked by a dotted line). Significance was accepted with *P < 0.05, **P < 0.01, ***P < 0.0005, ****P < 0.0001 for comparisons between treatment vs CTL (BSA 0,01g/mL) and $^{\#}$P < 0.05, $^{\#\#\#}$P < 0.0005, $^{\#\#\#\#}$P < 0.0001 for comparisons during time in the same group (24 and 6 h vs 1 h). Significance for additional fructose effect as accepted with $^{\$\$}$P < 0.01.
